# Simple, near, visual perception test for microsurgeon ‐Parallelism‐

**DOI:** 10.1002/cre2.684

**Published:** 2022-11-08

**Authors:** Tetsuya Hirata

**Affiliations:** ^1^ Research Group of iMage Assisted Precision Dentistry Osaka Japan

**Keywords:** microsurgeon, parallelism, visual perception

## Abstract

**Objectives:**

It is well known that a good microsurgeon needs eight important factors: a high resolution view, an optimally magnified view, optimal brightness of the working field, optimal working space, fine surgical instruments and devices, fine motor skills, precise hand−eye coordination, and fine visual perceptions. Of these factors, the first five are highly depending on manufacturer development abilities. The remaining factors have a lots of possibilities that microsurgeons can improve by themselves. A microsurgeon needs to identify shape, size, angle, inclination, length, height, depth, spatial position, centering in the optical field, orthogonality, and parallelism in a second. Knowing one's tendency and acuity in perceptions, learning perceptions that one is not good at, and paying selective attention on one's difficult perceptions, will provide better surgical outcome. Aim of this series of research is designing visual targets measuring specific visual perceptions for microsurgeons, achieving mean values of each perceptions, and identifying the tendency on each perceptions.

**Material and Methods:**

Two hundred and eighty volunteer dentists in Japan and France were tested and multiple comparisons were made among age, gender, visual acuity, three magnification levels, and inclination angles against a standard target.

**Results and Coclusion:**

There is a tendency that identifying 1° misalignment in parallelism is difficult.

## 1 INTRODUCTION

Successful microsurgical result is not the goal of surgery. Comparing with other's surgical result is not the main purpose of Microsurgery. To improve one's skill is the most important thing in Microsurgery and Microsurgery providing better outcome is understandable (Hirata, 2012). Optical systems/devices such as Microscopes, 3D video Microscopes, and so forth, is just one of the technology in extension/substitution of human faculties.

“Ability to look” consists of visual acuity, both‐eye motor function, and visual information processing function. Visual information processing function comprises shape perception, spatial perception, and hand−eye coordination.

The visual perception test in Neuropsychology (Goto et al., 2010; Yamadori, 1985) is made from length match task, size match task, position discrimination task, frequency doubling technology, judgment of line orientation, (A. Benton et al., 1975; A. L. Benton, 1978; A. L. Benton et al., 1983) visual discrimination task, and visual figure‐ground task. The visual function test is made from near visual acuity, field of vision, developmental eye movement test, inspection of convergence, saccade and eye tracking, functional acuity contrast test, stereo fly test, and color vision test. The visual recognition test is made of visual closure task and Kaufman assessment battery for children (K‐ABC).

Some of the tests mentioned above or other perception tests require specific projection equipment/systems and specific testing environment or field. Each microsurgeon works in a different environment or field such as in different room size, illumination, and visual systems. Microsurgeons need to know their visual perception tendency in a condition that is similar to their working places. Different equipment/systems do not give universal results. Microsurgeons perform microsurgery in 30−45 cm (12−18 inches) distance between one's forehead and fingers depending on their arm's length. Furthermore, microsurgeons sometimes work with their naked eyes and the other time, wear glasses or contact lens under each visual systems depending on individual visual acuity or convenience.

Neuropsychological tests to determine visual perception are designed with minimum 15° in inclination angle (usually 30°) because directional selectivity column in the visual cortex is alined every 15°. Discrepancy recognition of 15° (900 min, 81 mm, 900 pixels/282 dpi) is not suitable for microsurgery.

## 2 MATERIALS AND METHODS

Subjects were 280 volunteer dentists in Japan and France. Male 196 and female 84. Age 25−60 (20's: 10%, 30's: 40%, 40's: 30%, 50's: 10%, 60's: 10%). All were naive subjects. The procedures followed were in accordance with the ethical standards of the responsible committee on human experimentation and with the Helsinki Declaration of 1975, as revised in 2008. All study participants provided informed verbal consent before study enrollment.

Eliminating the influence of different projection equipment/systems, each set of visual targets were printed on individual A4 sized papers (282 dpi). The distance between these papers and subject's forehead was 300 mm like near visual acuity test chart. To avoid the influence of subject's head and body tilt, examination papers were set by themselves on an appropriate height table according to their receipting feeling of gravity force direction. Each subject sat on one's appropriate height chair comfortably.

It is said that visual function is largely influenced by eye movement. In central vision area, a human can see the target without moving one's eye. The size of the area is said to be that of 1° (60 min, 60 pixels/282 dpi).

**Figure 1:**
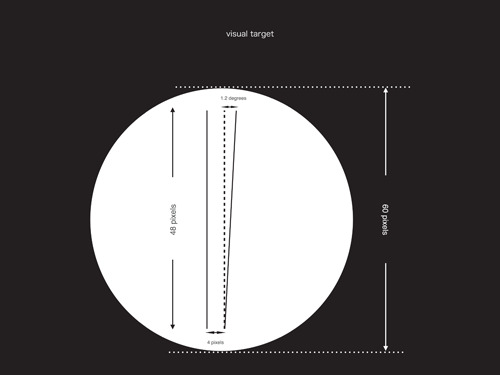
Visual target left is a standard target and right is a comparison target (282 dpi).

To minimize the influence of eye movement, the entire size of the simple, near, visual perception test for the microsurgeon on parallelism was a diameter 5.4 mm (60 min, 60 pixels/282 dpi) circle (Figure 1). The dominant eye was not tested.

Westheimer and McKee (1977) reported that shape and length of the visual target do not affect the result of the vernier vision test as long as the target is narrow in width. However, the distance between a standard visual target and a comparison visual target should be 2−4 min (0.18 mm, 2 pixels/282 dpi to 0.36 mm, 4 pixels/282 dpi).

Regarding the lengths of a line, the N1 amplitude showed some tendency to increase in the vertical dimension but decrease in the horizontal dimension (Ito, 2011). The adaptation of the human perception system to linear perspective images influences the visual perception (Walther et al., 2014). To eliminate length cognition assists parallelism perception, length of comparing visual targets and standard targets should be the same. The length of both a standard visual target and a comparison visual target was set at 4.5 mm (50 min, 50 pixels/282 dpi). To observe the effect between a 2D visual target and a line visual target, width of each targets was set at 4 pixels/282 dpi (0.36 mm, 4 min) and 1 pixel (0.09 mm, 1 min), respectively. The reason for using rectangle shape targets, which  were set at 4 pixels in width, was to minimize the influence of luminance perception. The color of the target was also set as black and the background was set as white for the same reason of minimizing the influence of color perception. The distance between a standard visual target and a comparison visual target was set as 4 min (0.36 mm, 4 pixels/282 dpi).

To examine the parallelism against the vertical target, a standard target inclination of 0° was set. A comparison visual target of 0° was also set, 1°, 2°, and 3° against the bottom edge of printing papers were stablished. Inclination were set for both right and left direction. When a comparison target inclined at 1°, 2°, and 3° against the bottom edge of printing papers to the left direction was used, a comparison target was placed at the left side of a standard target. When a comparison target inclined at 1°, 2°, and 3° against the bottom edge of printing papers to the right direction was used, a comparison target was placed at the right side of a standard target. A total of seven conditions for three different degrees were prepared. To examine the parallelism against an oblique target, a standard target inclination set at 1°, 2°, and 3° against the bottom edge of printing papers for both right and left direction were used. A comparison target, which was inclined with the same amount of angulation in both the same direction and the opposite direction, was used. A total of four conditions for three different degrees were prepared (right way parallel target, left way parallel target, v shape target, and inverted v shape target).

Although the size of the visual target exceeded the size of the central vision area (1°, 60 min, 60 pixels/282 dpi), to investigate parallelism perception among naked eyes, under loupe usage and under operating Microscope usage, each sets of targets were printed on each A4 sized papers at two times (designed for 2 × 2 = 4 times loupe) and at four times (designed for 4 × 4 = 16 times operating Microscope) at the same time, and they were also tested. To eliminate the influence of different loupe systems or operating Microscope systems, which have different illumination, magnification, resolution, focal length, and convergence angle or stereo base length, in addition to eliminating the influence of individual difficulty in loupes or operating Microscopes usage because of each organ position, alignment, or discrepancy, tests were performed on the magnified printed targets; they were not tested on unmagnified printed targets under loupes or operating Microscopes.

It is well known that the longer the targets are observed, the more perception factors arise. Human visual line keeps moving and it is difficult to hold it at one position for a long time (max 50 ms). However, a microsurgeon is accustomed to working under these involuntary eye movement. Furthermore, a standard target needs to cognize first under this comparison task. It is said that it takes 1 s. Presentation time of each sets of targets was set at 2 s.

Before performing simple, near, visual perception test for each microsurgeon on parallelism, a near visual acuity test chart was taken.

Statistical analysis was performed by one‐way ANOVA using post hoc Tukey HSD test calculator for comparing multiple treatments.

## 3 RESULT

Accuracy of this simple, near, visual perception test for microsurgeon on parallelism was specificity 91%, sensibility was 98%, positive predictive value was 91%, and negative predictive value was 98% (Sherman et al., 2011).

Some subjects showed some preferences in the shape of the target: one showed better perception for line shape targets than rectangle shape targets, while other showed better perception for rectangle shape targets than line shape targets. Some subjects showed some preferences in the inclination direction: one showed better perception for left direction than right direction, while other showed better perception for right direction than left direction. However, those who had preferences in inclination direction did not have discrepancy in right and left visual acuity. There were not any significant differences among age, gender, visual acuity, shape of target, and inclination direction (right or left).

**Figure 2:**
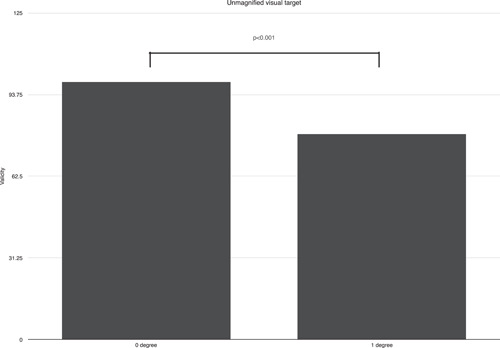
Validity at unmagnified visual target validity of 0 and 1° inclined comparison targets against vertical standard target were compared (*p* < .001).

At the naked eye, when unmagnified targets were used, both a standard visual target and a comparison visual target set at vertical direction, parallel to gravity, 0° against the bottom edge of printing papers in this study, showed mean value validity of 98.57%. At the naked eye, a standard visual target set at 0° against the bottom edge of printing papers and a comparison visual target set at 1° inclined against the bottom edge of printing papers toward either right and left direction showed mean value validity of 78.75%. Between these two situations, significant differences were found, *p* < .001 (Figure 2).

As expected, since magnified visual targets exceed the size of central vision area, research performed with magnified targets showed significantly worse mean value validity compared to test performed with unmagnified targets. However, at both two times and four times magnified targets, both a standard visual target and a comparison visual target set at 0° against the bottom edge of printing papers showed mean value validity of 97.14% and 95.71%.

**Figure 3:**
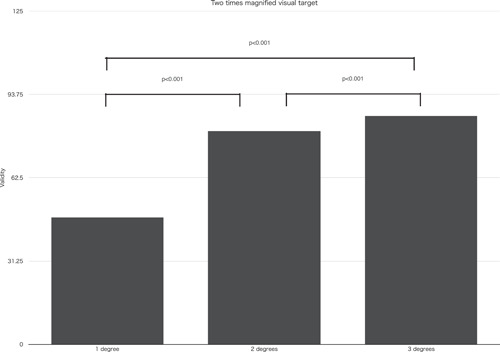
Validity at two times magnified visual target, validity of 1°, 2°, and 3° inclined comparison targets against vertical standard target were compared (*p* < .001).

**Figure 4:**
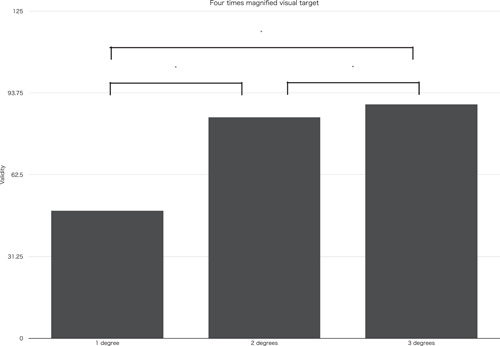
Validity at four times magnified visual target, validity of 1°, 2°, and 3° inclined comparison targets against vertical standard target were compared (*p < .05).

When magnified targets were used, significant differences was found between two times (designed for 2 × 2 = 4 times loupe usage) and four times (designed for 4 × 4 = 16 times operating Microscope usage) magnified targets at the following condition. When a standard visual target was set at 0° against the bottom edge of printing papers and a comparison visual target was set at 1 and 2° inclined against the bottom edge of printing papers toward either right and left direction, it showed significant differences, *p* < .01 and *p* < .01. Moreover, significant differences were found among a comparison visual target set at 1°, 2°, and 3° inclined against the bottom edge of printing papers toward either right and left direction and a standard visual target set at 0° against the bottom edge of printing papers in both two times and four times magnified targets. When two times magnified targets were used, *p* < .001 (Figure 3) and when four times magnified targets were used, *p* < .05 (Figure 4). At two times magnified targets with a comparison visual target set at 1° inclined against the bottom edge of printing papers toward either right and left direction and a standard visual target set at 0° against the bottom edge of printing papers, mean value validity was 47.50%. At four times magnified targets with a comparison visual target set at 1° inclined against the bottom edge of printing papers toward either right and left direction and a standard visual target set at 0° against the bottom edge of printing papers, mean value validity was 48.75%.

## 4 DISCUSSION

As long as the entire size of simple, near, visual perception test for microsurgeon on parallelism is within diameter 5.4 mm (60 min, 60 pixels/282 dpi) circle, this test is reliable. For comparing perception between magnified and unmagnified situation, the visual target should be reduced in size.

When either magnified or unmagnified targets were used, both two line or two rectangle targets were set in the vertical direction, parallel to gravity, 0° against the bottom edge of printing papers in this study, mean value validity was 98.57%, 97.14%, and 95.71%. Vertical and horizontal recognition refers to gravity. This perception that refers to gravity seems to overcome other perceptions. When considering the influence of luminance or color perception for the rectangle target at the same time, wider width target should be prepared. Furthermore, whether magnified or unmagnified targets were used, a comparison target set at 1° inclined against vertical standard target case showed mean value validity 78.75%, 47.50%, and 48.75%. Significant differences were *p* < .001, *p* < .01, and *p* < .01. In visual perception of parallelism, there is a tendency that recognizing 1° misalignment is difficult.

Cognizing parallelism toward two line or two rectangle targets seems to utilize distance between two subjects and angle of two subjects against gravity force direction. Parametric multiple comparisons among the result of simple, near, visual perception test for microsurgeon on parallelism, angle and length are needed to report after presenting each results. At the same time, assimilation (decrease of differences) and contrast (increase of difference) (Goto et al., 2007) that are seen in length illusion needs to be considered since parallelism perception influenced more with length perception than angle perception.

There are many studies regarding whether visual learning effect is maintained for long time or disappears in a short period. It is reported that using the directional discrimination threshold task, a learning effect persists for 1 and 1/2 years and in a passive visual perceptual learning, learning effect maintains at least 4−6 months after last visual stimuli (Karni & Sagi, 1993; Watanabe et al., 2002).

It is reported that paying selective attention, how much degree of concentration, on the task affects the result depending on the amount of attention. The more attention, the better result (Lyamzin et al., 2021).

## 5 CONCLUSION

1. This simple, near, VPTM for parallelism is reliable. Specificity is 91%, sensibility is 98%, positive predictive value is 91%, and negative predictive value is 98%.
2. When both the two visual targets are lined in vertical direction, it means along with gravity force direction, parallelism perception works well.
3. There is a tendency that identifying 1° misalignment in parallelism is difficult.

## AUTHOR CONTRIBUTIONS

Not Applicable.

## CONFLICTS OF INTEREST

The author declares no conflict of interest.

## Data Availability

Data that support the findings of this study are available from the corresponding author upon reasonable request.
